# Parametric Study of Proton-Capture Nucleosynthesis of Al27 via the Extended Ne-Al Chain

**DOI:** 10.21203/rs.3.rs-9495756/v1

**Published:** 2026-04-29

**Authors:** Deva Pratim Mahanta, Apurba Talukdar, Swapnajyoti Sarma, Mrinmay Medhi, Dhanjit Talukdar, Upakul Mahanta, Arup Bharali

**Affiliations:** 1 Bhattadev University

**Keywords:** Nucleosynthesis, H-burning, Metallicity, Abundances

## Abstract

We present a parametric investigation of proton-capture reactions within the extended Neon–Sodium–Magnesium–Aluminum (Ne-Al) nuclear reaction network, with particular emphasis on the production of Al27. The study explores the sensitivity of aluminum synthesis to temperature conditions in the range $T_{9}=0.02-0.10$ at a fixed density of 100 g cm^−3^). The calculations are performed using a single-zone nuclear reaction network with simplified initial abundance assumptions, including an idealized uniform distribution of selected isotopes to probe reaction pathways. The nuclear network is solved numerically to obtain the evolution and quasi-equilibrium mass fractions of the involved species. The results demonstrate that the extended chain Ne-Al can efficiently produce Al27 within the temperature range explored, with the final abundances showing a systematic dependence on the temperature and reaction flow characteristics. A statistical comparison with representative observational abundance datasets yields a correlation coefficient exceeding 0.85 and a mean absolute deviation (MAD) of 0.40 dex, indicating that the model captures general abundance trends despite its simplified assumptions. This work does not incorporate a full stellar evolution model or transport processes such as mixing and diffusion. Therefore, the results should be interpreted as indicative of nuclear reaction pathways and sensitivities, rather than direct predictions of stellar surface abundances. The study provides insight into the role of proton-capture processes in shaping intermediate-mass element synthesis under controlled thermodynamic conditions.

## 1 Introduction

Early A-type and late B-type stars generally occupy an effective temperature range in which photospheric mass motions are expected to be minimal. In contrast to chemically peculiar (CP) stars, they lack surface anomalies and, under LTE analyses, display chemical abundances closely resembling solar values, with no evidence of periodic or aperiodic variability (Wolff and Preston (1978)). The occurrence of such normal stars within the temperature domain typical of CP stars, however, appears to be uncommon. Spectral peculiarities are reported in approximately 20% of A-type stars (Cowley et al. (1969)), and CP stars are estimated to comprise at least one-third, and possibly a significantly larger fraction, of the total A- and B-type stellar population. Within this broader category of CP stars, HgMn stars represent a distinct subgroup confined primarily to the late B-type domain (B7–B9), near the early A-type boundary (Preston (1974); Adelman (1994)). Their defining characteristic is the pronounced overabundance of elements such as mercury and manganese (Smith (1996)), in contrast to the near-solar compositions of their normal late B- and early A-type counterparts.

Thus, HgMn stars provide a striking example of how chemical peculiarity manifests within a temperature regime otherwise populated by apparently chemically normal stars. Further, the view that normal, sharp-lined A- and B-type stars represent the majority may itself be misleading, as such stars may actually be exceptions rather than the rule (Ramella et al. (1989); Lemke (1990)). HgMn stars, being main-sequence B-type stars of luminosity class V, occupy surface temperature ranges from about 1 × 10^4^ to 3 × 10^4^ K (Habets and Heintze (1981)). Like other B-type stars, they are highly luminous and blue, with spectra showing neutral helium (most prominent at the B2 subclass), moderate hydrogen lines, and weak or absent metallic features. These stars are generally young and evolving rapidly through the early main-sequence phase, with metallicities comparable to solar values and stellar winds reaching velocities of about 3 × 10^3^ km/s (Aschenbach et al. (1998)). In addition, their heavy elements often display non-terrestrial isotopic abundances, further highlighting their chemical anomalies. Another remarkable property of HgMn stars is their strong association with binarity. Early radial velocity surveys already suggested that many HgMn stars belong to binary or multiple systems (Wolff and Preston (1978) & Preston (1974)), and more recent spectroscopic and high-resolution interferometric studies have confirmed that multiplicity among HgMn stars is exceptionally high, possibly exceeding 80–90% (Schöller et al. (2010); Chojnowski et al. (2020)). Well-studied systems such as 41 Eridani illustrate how HgMn stars frequently reside in close binaries (Hummel et al. (2017)), while cases like HD 161701, which pairs an HgMn star with an Ap companion, underscore the central role of binarity in shaping the observed peculiarities (Gonzalez et al. (2014)). These findings suggest that the binary environment provides favorable conditions—such as reduced rotational mixing, tidal effects, and possible mass transfer episodes—for the operation of atomic diffusion and the emergence of extreme surface abundance anomalies.

It is well established that the surface abundance anomalies observed in HgMn stars arise primarily due to atomic diffusion processes such as radiative levitation and gravitational settling, rather than in-situ nuclear burning. Therefore, the present study does not attempt to directly model observed surface abundances of HgMn stars. Instead, it explores nuclear reaction pathways that may contribute to isotopic abundance variations under controlled thermodynamic conditions. Such parametric nuclear network studies are useful for isolating the role of specific reaction chains and for understanding sensitivity to physical conditions, even in the absence of full stellar evolution modeling.

Although HgMn stars are frequently found in binary systems and their surface abundance anomalies are primarily governed by atomic diffusion, the investigation of nuclear reaction pathways remains relevant at a more fundamental level. In particular, a parametric, single-zone treatment of the extended Ne-Al chain provides a controlled framework for isolating the efficiency and sensitivity of Al27 production under specified thermodynamic conditions. While such an approach does not capture the structural complexity of binary stellar evolution or the transport processes that determine photospheric abundances, it allows one to quantify the intrinsic nuclear physics governing isotope production. This is especially useful for identifying dominant reaction channels, assessing temperature-dependent behavior, and evaluating the impact of reaction rate uncertainties. In this sense, the present model does not attempt to reproduce the observed surface abundances of individual systems, but instead provides a baseline for understanding whether proton-capture processes within the Ne-Al chain can efficiently generate Al27 under conditions that may plausibly occur in stellar interiors, including those influenced by binary interaction. Such parametric studies therefore serve as an essential first step toward more comprehensive models that couple nucleosynthesis with stellar structure and transport phenomena.

Against this background, the role of nuclear processes becomes relevant in a more limited and exploratory sense. Nucleosynthesis involving the Ne-Na and Mg-Al cycles has been discussed extensively (Mahanta et al. (2017); Angulo et al. (1999)), and recent experimental determinations of reaction cross sections (Iliadis et al. (2010)) provide opportunities for refined analysis of reaction pathways. In this context, the production of Al27 is of particular interest, as aluminium isotopes serve as useful tracers of nucleosynthetic processes. The ratio of Al26 to Al27, for example, provides insights into nuclear reaction flows and isotope production under different thermodynamic conditions (Roriz et al. (2023)).

In the present work, we investigate an extended Ne-Al reaction network that differs from the earlier framework of (Mahanta et al. (2023)) in two key respects: (i) the inclusion of a more complex proton-capture network with a branching point at Na23 (Table 1), and (ii) the adoption of simplified initial conditions designed to systematically explore reaction sensitivities. The principal objective of this study is to examine the production of Al27 within a parametric, single-zone framework, rather than to model specific stellar environments.

Section 2 outlines the adopted physical conditions and assumptions. Section 3 introduces the extended Ne-Al reaction chain and discusses its structure. Sections 4 and 5 present the mathematical formulation and computational methodology used to derive elemental abundances. Section 6 addresses sources of uncertainty, while Section 7 presents the numerical results. Finally, Section 8 discusses the implications of the results within the limitations of the adopted model and provides concluding remarks. Section 9 gives the declaration about this research.

## 2 The Stellar Situation

Since the pioneering discovery of a binary star by the English astronomer William Herschel (1738–1822) in the late 18th century, the study of stellar multiplicity has advanced considerably. A survey of 123 nearby Sun-like stars conducted about a decade ago revealed that 57% host one or more stellar companions (Pattnaik et al. (2011)). More recent statistical analyses have shown that, among stars completing their nuclear evolution within a Hubble time, the binary fraction increases with mass—from approximately 40–60% for solar-mass stars to nearly 100% for more massive A-, B-, and O-type stars (Postnov and Yungelson (2014)). These results suggest that binarity is the norm rather than the exception: most stars are born, and evolve, as members of binary or higher-order multiple systems. Consequently, the evolutionary pathways of binary stars, particularly with regard to their elemental abundance patterns, often diverge significantly from those of isolated single stars due to mutual gravitational interactions.

In this work, we consider stars undergoing binary interaction through Roche lobe overflow (RLOF). This mechanism carries several evolutionary consequences, including truncated evolutionary phases and mass accretion episodes. During accretion, material-protons in particular-can be transferred from the companion star and participate in various nuclear burning processes. If accretion is sufficiently rapid, a common envelope (CE) may form around the binary (Paczynski (1976)). We assume that CE evolution proceeds on timescales short compared to surface nuclear burning, and that the infalling material does not disrupt the hydrostatic equilibrium of the accreting star. Under these conditions, the stellar temperature may be treated as effectively constant. The processed material produced in these circumstances can be expelled directly into the interstellar medium (ISM), since RLOF does not necessarily proceed conservatively. Such ejected matter may subsequently be captured by other stars, including the original companion. In the present study, we do not address the detailed physical mechanisms driving mass loss or the selective enrichment of H-burning products. Rather, we focus on one possible outcome of binary interactionnamely, the nucleosynthetic processing of accreted material and its potential role in producing the observed chemical peculiarities associated with hydrogen-burning products.

## 3 The Extended Ne-Al Chain: Nuclear Transformation Pathways

The neon–sodium (Ne–Na) cycle of hydrogen burning enables the conversion of protons into helium with neon and sodium isotopes acting as catalysts. Following carbon exhaustion, stellar cores contract, raising temperature and density until neon burning ignites at about 10^9^ K. Although the Ne–Na cycle contributes little to stellar energy production, it plays a key role in the synthesis of elements between Ne20 and Mg24 (Marion and Flower (1957)).

- Ne20(p,γ)21Na:
  This reaction represents the bottleneck of the Ne–Na cycle, as its comparatively low rate strongly influences the nucleosynthesis of Ne, Na, and Mg isotopes, while also contributing to hydrogen burning in various stellar environments, including red giants, asymptotic giant branch (AGB) stars, massive stars such as B-type stars, and ONe novae. Within the relevant temperature range for these sites ($T_{9}=0.05-0.5$, where $T_{9}$ is in units of 10^9^ K), the reaction rate is governed primarily by direct proton capture, along with contributions from the high-energy tail of a sub-threshold resonance at $E_{x}=2425\;\text{keV}$ in the Na21 compound nucleus (Mahanta et al. (2023)).
- Ne21(p,γ)22Naβ+,νe22Ne:
  ^22^Ne is formed via the decay of radioactive ^22^Ne; however, at temperatures $T_{9}>0.07$, proton capture on ^22^Na dominates over β-decay, effectively bypassing ^22^Ne in the Ne–Na cycle. Importantly, ^22^Ne serves as a seed nucleus for subsequent proton captures, linking the Ne–Na cycle to the extended Ne–Al chain and enabling the synthesis of heavier nuclei such as ^27^Al (Mahanta et al. (2023)).
-
Ne22(p,γ)23Na:

  This reaction is likely the most uncertain in the entire Ne-Na cycle, primarily due to the presence of multiple resonances at stellar energies (Depalo et al. (2015)). Experimental investigations over the 70–360 keV range using γ-ray spectroscopy (Görres et al. (1982)) indicate that additional resonances in ^23^Na within this energy window may significantly affect stellar reaction rates, particularly at $T_{9}\leq 0.5$. At temperatures above $T_{9}=0.05$, hydrogen burning can proceed efficiently through ^23^Na production, which is consistent with the temperature conditions assumed in our study.
-
Na23(p,γ)24Mg&Na23(p,α)20Ne:

  The competition between (p,γ) and (p,α) channels determines whether material remains trapped within the Ne–Na cycle or is processed onward to Mg and Al (Hale et al. (2004)). The Na23(p,γ)24Mg rate is mainly governed by direct capture and low-energy resonances, with its uncertainty significantly reduced by the improved reaction rates of (Iliadis et al. (2010)) in the temperature range $T_{9}=0.02-0.1$, as noted by (Cessaratto et al. (2013)). Within our assumed temperature conditions, this provides a reliable estimate. By contrast, the competing Na23(p,α)20Ne channel does not close a cycle but instead replenishes Ne abundances in the reaction network.
- Mg24(p,γ)25Al:
  The Mg–Al cycle becomes active in the deepest layers of an H-burning zone once the temperature exceeds $T_{9}>0.04$ (Piersanti et al. (2013); Powell et al. (1999)) demonstrated that ^24^Mg and ^25^Mg are converted into Al at $T_{9}=0.02-0.06$ within the hydrogen-burning shells of RGB stars. The key reaction, Mg24(p,γ)25Al, exhibits a resonance at 222 keV (Craig (1956); Trautvetter (1975)) and is particularly important, as ^24^Mg depletion is observed in Al-rich stars (Denissenkov and VandenBerg (2003)). This makes the reaction highly feasible within the temperature range considered in our study.
- M25g(p,γ)26gAl:
  This reaction is a crucial component of the Mg–Al cycle, as it links ^25^Mg burning to the synthesis of ^26^Al, a radioactive isotope with significant astrophysical implications. The production of Al26g occurs under H-burning conditions, particularly in stellar shells where $T_{9}>0.04$, and contributes directly to the observed depletion of Mg and enrichment of Al in evolved stars. (Limata et al. (2010)) highlighted the presence of a strong resonance at 304 keV, confirming that the reaction proceeds efficiently at stellar temperatures $\left(T_{9}\geq 0.03\right)$, thereby sustaining the Mg–Al cycle.
- Mg25(p,γ)26gAl:
  This reaction represents one of the key steps in the Mg-Al cycle, as it governs the conversion of ^25^Mg into ^26^Al under hydrogen-burning conditions. The Mg25(p,γ)26gAl pathway remains active in stellar H-shell environments at temperatures $T_{9}>0.04$, providing an efficient channel for aluminum enrichment while simultaneously depleting magnesium. A particularly important feature of this reaction is the presence of a strong resonance at 304 keV, which becomes significant at stellar temperatures as low as $T_{9}\geq 0.03$ (Limata et al. (2010)). This resonance enhances the reaction rate within the temperature regime relevant to main-sequence and evolved stars, ensuring that ^26^Al production is robust in astrophysical sites where the Mg–Al cycle operates. The synthesis of ^26^Al is of considerable astrophysical interest, not only because it contributes to the chemical anomalies observed in Mg-depleted, Al-rich stars, but also because its radioactive decay (with a half-life of ~ 7.2 × 10^5^ years) produces the characteristic 1.809 MeV γ-ray line, directly detected in the interstellar medium and widely regarded as evidence of ongoing nucleosynthesis in the Galaxy. Thus, the Mg25(p,γ)26gAl reaction provides both a critical link in the Mg-Al cycle and a direct observational tracer of stellar nucleosynthesis.
- Al27(p,α)24Mg:
  The isotopic composition of magnesium in the interstellar medium (ISM) is widely recognized as a sensitive probe of star formation and chemical enrichment processes across cosmological timescales. Variations in the relative abundances of ^24^Mg, ^25^Mg, and ^26^Mg provide important constraints on the cumulative nucleosynthetic contributions of successive stellar generations. Within this framework, the reaction responsible for driving the destruction of ^27^Al and the concomitant production of ^24^Mg plays a pivotal role during stellar hydrogen burning. Palmerini et al. (2021) demonstrated that this process becomes particularly efficient at temperatures of $T_{9}\approx 0.055$, characteristic of H-burning shells in evolved stars as well as in certain massive main-sequence environments. At these temperatures, the competition between proton captures and subsequent decay pathways regulates the relative yields of Mg and Al isotopes, directly influencing the observed Mg isotopic ratios in the ISM. In the context of our study, the temperature regime identified by Palmerini et al. (2021) is in excellent agreement with the values assumed in our calculations, further supporting the astrophysical plausibility of the proposed nucleosynthesis pathway. Thus, the extended Ne-Al chain is as follows:

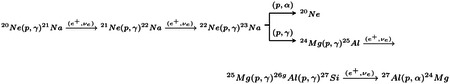



## 4 Hydrogen burning nucleosynthesis:

The rate of nuclear reactions is dependent on the density of the reactants, the velocity of one reactant relative to another, and the probability of a reaction occurring Mahanta et al. (2023). Mathematically,

(2) $$R=\frac{\rho^{2}N_{A}}{A_{H}A_{i}}\left[X_{H}X_{i}\left(N_{A}<\sigma V>\right)\right]$$

where $N_{A}<\sigma V>$ is the reaction rate constant and $X_{i}$ is the mass fraction of any other heavy element. From this, the lifetime of proton capture can be calculated. Here, the required reaction rate constants are taken from Iliadis et al. (2010) and are recommended medium reaction rate values for Ne20(p,γ)21Na,Ne21(p,γ)22Na,Ne22(p,γ)23Na,Na23(p,γ)24Mg,Na23(p,α)20Ne,Mg24(p,γ)Al25,Mg25(p,γ)26gAl,Mg26(p,γ)27Al,Al27(p,α)24Mg. Table 2, Table 3 and Table 4 shows the calculated values for the proton capture lifetimes for various elements for γ-decay, α- decay and n-decay respectively under stellar conditions.

## 5 Evolution of elemental abundance:

The generalized differential equation that governs the evolution of any element in terms of number density via a proton-capture reaction or a β-decay, or both, at the enhanced condition (Mahanta et al. (2023)) is given by,

(3) $$\frac{\text{d}N_{i}}{\text{d}t}=-N_{i}N_{H}\langle \sigma v\rangle_{p,i}+N_{j}N_{H}\langle \sigma v\rangle_{H,j}\pm \lambda_{k}N_{k}$$

where $\lambda_{k}$ is the decay constant of an unstable nucleus with number density $N_{k}$. As the chain involves proton-capture reactions and β-decays, the abundances will primarily depend upon the lifetime of these processes. If the β-decay lifetime $\tau_{\beta }$ for an unstable element in the cycle is shorter than the proton-capture lifetime $\tau_{p}$ for the same element, then the β-decay lifetime can be bypassed and thus the element can be thought of as representing the next stable element in the chain having the same mass number. Considering that Equation (3) for the reaction chain Ne20(p,γ)21Na,Ne21(p,γ)22Na,Ne22(p,γ)23Na,Na23(p,γ)24Mg,Na23(p,γ)20Ne,Mg24(p,γ)25Al,Mg25(p,γ)26gAl,Mg26(p,γ)Al27,Al27(p,α)24Mg takes the form of the following differential rate equations in 5.

The following equation can be expressed as a function of the hydrogen mass fraction to get a series of first-order simultaneous linear differential equations (Mahanta et al. (2023)) as

(5) $$\frac{\text{d}X_{i}}{\text{d}X_{H}}=\frac{\left(-R_{p,i}X_{i}+\frac{A_{i}}{A_{j}}R_{p,j}X_{j}\right)}{-\left[\sum_{A_{i}}^{A_{j}}\;\;\left(\frac{1}{A_{i}}R_{p,i}X_{i}\right)\right]}$$

which are then solved for the suitable initial conditions. Here, $R_{p,i}\;\text{s}$ are $\left[N_{A}\langle \sigma v\rangle \right]$ terms for the respective proton-capture reactions. $A_{i}$ and $A_{j}$ stand for mass numbers of different nuclei.

## 6 Abundance Uncertainties and Statistical Assessment

The thermonuclear reaction rates used in our calculations were adopted from the Monte Carlo framework of Iliadis et al. (2010), which yields statistically robust and physically meaningful estimates of reaction rate uncertainties. In this method, the recommended rate is defined as the median of the probability density function (0.50 quantile), while the low and high rates correspond to the 0.16 and 0.84 quantiles, respectively, representing a 68% confidence interval. This probabilistic treatment accounts for uncertainties in a statistically consistent manner, rather than as fixed systematic offsets, and allows for more realistic error propagation in stellar models. In this study, we applied this prescription by performing calculations with both the low and high reaction rate limits for each relevant channel, thereby quantifying the sensitivity of equilibrium mass-fraction abundances to nuclear physics uncertainties and strengthening the reliability of our results.

To evaluate the reliability of the derived abundances, we conducted a statistical analysis using the Pearson correlation coefficient and the mean absolute deviation (MAD). The Pearson coefficient, calculated between the observed values reported by Smith (1993) and the estimates obtained in this work, exceeded 0.85, indicating a strong correlation. The MAD, found to be 0.4 dex, quantified the average deviation of individual measurements from the mean and thus provided a robust measure of the intrinsic spread within the sample.

## 7 Calculation of abundances:

The system of eight coupled first-order linear differential equations, analogous to Eq. (6), was numerically integrated at different temperatures to model the evolution of the extended Ne-Al reaction network. The calculations were performed for a

(4) $$\frac{{\text{d}}^{20}Ne}{\text{d}t}=-\frac{{\text{d}}^{20}Ne}{\tau_{20}}+\left(1-B_{r}\right)\frac{{\text{d}}^{23}Na}{\tau_{23}}\;\frac{{\text{d}}^{21}Ne}{\text{d}t}=-\frac{{\text{d}}^{21}Ne}{\tau_{21}}+\frac{{\text{d}}^{20}Ne}{\tau_{20}}\;\frac{{\text{d}}^{22}Ne}{\text{d}t}=-\frac{{\text{d}}^{22}Ne}{\tau_{22}}+\frac{{\text{d}}^{21}Ne}{\tau_{21}}\;\frac{{\text{d}}^{23}Na}{\text{d}t}=-\frac{{\text{d}}^{23}Na}{\tau_{23}}+\frac{{\text{d}}^{22}Ne}{\tau_{22}}\;\frac{{\text{d}}^{24}Mg}{\text{d}t}=-\frac{{\text{d}}^{24}Mg}{\tau_{24}}+B_{r}\frac{{\text{d}}^{23}Na}{\tau_{23}}+\frac{{\text{d}}^{27}Al}{\tau_{27}}\;\frac{{\text{d}}^{25}Mg}{\text{d}t}=-\frac{{\text{d}}^{25}Mg}{\tau_{25}}+\frac{{\text{d}}^{24}Mg}{\tau_{24}}\;\frac{{\text{d}}^{26g}Al}{\text{d}t}=-\frac{{\text{d}}^{26g}Al}{\tau_{26}}+\frac{{\text{d}}^{25}Mg}{\tau_{25}}\;\frac{{\text{d}}^{27}Al}{\text{d}t}=-\frac{{\text{d}}^{27}Al}{\tau_{27}}+\frac{{\text{d}}^{26g}Al}{\tau_{26}}$$

hydrogen mass fraction varying down to $X_{H}=0.6$, in order to trace the approach to quasi-equilibrium abundances under controlled thermodynamic conditions. The resulting equilibrium mass fractions were obtained across the temperature range $T=(0.02-0.1)\times 10^{9}\;\text{K}$.

The adopted temperature range spans regimes that overlap with hydrogen-burning conditions and extend slightly beyond them. This interval is not intended to represent a single astrophysical site, but rather to explore the sensitivity of reaction pathways across a controlled range of thermal conditions. The lower bound $\left(T_{9}\sim 0.02\right)$ corresponds to the onset of efficient proton-capture reactions, while the upper bound $\left(T_{9}\sim 0.1\right)$ allows enhanced activation of the Ne-Al chain without entering regimes characteristic of advanced burning stages such as neon burning. This choice enables a systematic investigation of temperature-dependent reaction flows and facilitates the identification of dominant pathways and bottlenecks within the network.

Such temperature-density conditions are broadly representative of hydrogen-burning environments in stellar interiors, including hydrogen-burning shells in red giant branch (RGB) and asymptotic giant branch (AGB) stars, as well as deeper layers of more massive stars (Iliadis (2015); Kippenhahn et al. (2012)). Under these conditions, the Ne-Na and Mg-Al cycles are known to operate efficiently and influence the isotopic composition of intermediate-mass elements.

In the context of HgMn stars, whose observed surface abundance anomalies are primarily governed by atomic diffusion processes, these temperature regimes are not directly realized in their photospheric layers. Nevertheless, the present calculations remain relevant in an indirect sense, as they characterize the nuclear production of isotopes such as Al27 in stellar interiors or in earlier evolutionary stages. Such nucleosynthetic signatures may subsequently be modified and redistributed through diffusion, radiative levitation, or binary-related processes, which are known to play a dominant role in shaping observed surface abundances (Michaud (1970); Alecian (2015)). Accordingly, the adopted temperature range should be interpreted as representative of underlying nucleosynthetic environments rather than the observable layers of these stars.

The initial abundances adopted in this study are simplified and do not reflect realistic stellar compositions. In particular, an equal distribution among isotopes has been assumed for exploratory purposes. We acknowledge that this is not representative of astrophysical conditions, where abundances typically follow solar or metallicity-dependent distributions. However, this assumption is intentionally adopted to establish a controlled baseline in which no isotope is preferentially enhanced at the outset. This facilitates a transparent analysis of the intrinsic behavior of the nuclear reaction network, allowing clearer identification of dominant reaction channels, branching points, and temperature-dependent sensitivities. The adopted initial condition should therefore be regarded as a diagnostic tool rather than a physically realistic input.

Accordingly, the initial heavy-element mass fraction was equally distributed among the isotopes ^20^Ne, ^21^Ne, ^22^Ne, ^23^Na, ^24^Mg, ^25^Mg, ^26^Mg, and ^27^Al. The baseline composition was taken as $X_{H}=0.70,X_{He}=0.2995$, and $X_{Z}=0.0005$, where the heavy-element fraction $X_{Z}$ is equally partitioned among the eight isotopes (XNe20=XNe21=XNe22=XNa23=XMg24=XMg25=XMg26=XAl27=0.0000625), ensuring mass conservation such that $X_{H}+X_{He}+X_{Z}=1$.

**Fig. 1:**
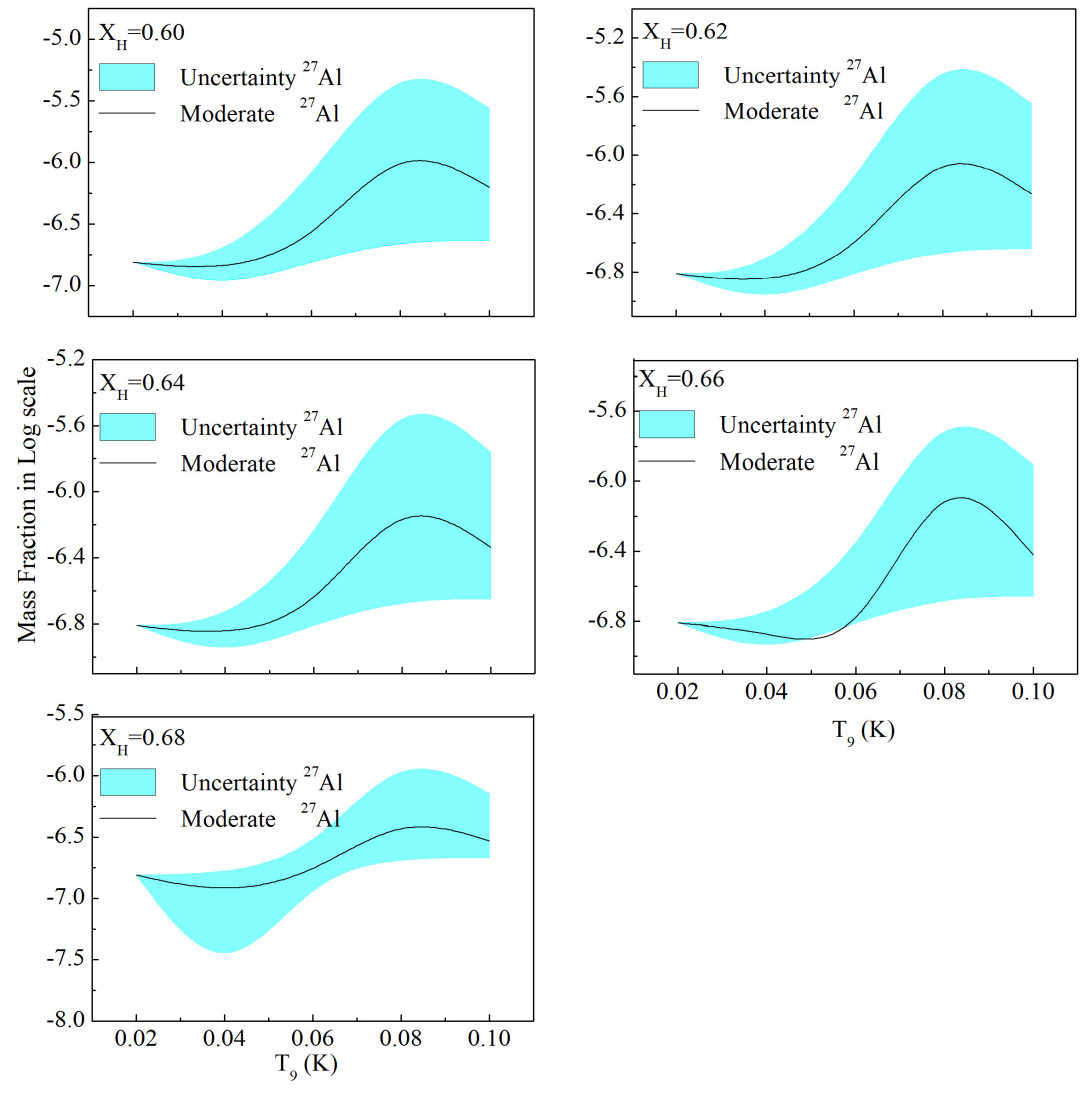
The abundances of Al27 is shown as a function of temperature (T_9_) at different hydrogen mass fractions $\left(X_{H}\right)$ (top left at $X_{H}=0.60$, top right at $X_{H}=0.62$, middle left at $X_{H}=0.64$, middle right at $X_{H}=0.66$, and bottom left at $X_{H}=0.68$).

The choice of a low heavy-element fraction is consistent with the aim of examining nucleosynthesis in a simplified environment where the influence of seed nuclei is minimized. While reduced metallicity does not alter the fundamental nuclear reaction mechanisms, it affects the efficiency and progression of reaction cycles by limiting the initial abundance of participating isotopes. In stellar contexts, lower metallicity is also associated with reduced opacity, leading to higher internal temperatures and more compact structures (Ekström (2021)). Although these structural effects are not explicitly modeled here, the adopted composition provides a reasonable baseline for exploring how nuclear processes respond under such conditions. The computed quasi-equilibrium abundances of the stable isotopes, expressed as mass fractions at different values of the hydrogen mass fraction, are summarized in Table 5. The corresponding variation of abundances with temperature, shown on a logarithmic scale, is presented in Figure 1. These mass fractions are subsequently used to derive abundance ratios of the stable isotopes of a given element (X) relative to Fe using the formalism described in (Mahanta et al. (2023)).


(6) $$\varepsilon =\left(\frac{X}{H}\right)+6.17$$

These calculated values of abundances are then compared with literature values taken from Smith (1993), for only those fractions of stars for which the [Fe/H] values are available (in SIMBAD database). Table 6 is the comparative tables against these estimated values of Al.

## 8 Discussion and Conclusion

In the present study, we have carried out a parametric investigation of the synthesis of intermediate-mass nuclei through proton-capture reactions within the extended Ne-Al reaction network, employing updated thermonuclear reaction rates (Iliadis et al. (2010)). The network calculations were performed under controlled thermodynamic conditions, with a fixed density of 100 g cm^−3^ and a temperature range $T=(0.02-0.1)\times 10^{9}\;\text{K}$, in order to examine the sensitivity of ^27^Al production to reaction flow dynamics. The adopted density corresponds to a relatively low-density stellar environment and is chosen to ensure that nucleosynthesis proceeds through standard thermonuclear reaction mechanisms. Within this regime, density is not a strongly sensitive parameter for the proton-capture pathways considered, and the reaction flow is primarily governed by temperature and reaction rate uncertainties. Only at significantly higher densities (≳ 10^5^ g cm^−3^) would pycnonuclear effects become important, leading to qualitatively different reaction dynamics.

Accordingly, the present calculations are not intended to represent a specific stellar object, but rather to provide a controlled framework for examining the efficiency and behavior of the extended Ne-Al chain under representative thermodynamic conditions. The results demonstrate that the synthesis of ^27^Al and related nuclei proceeds predominantly through thermonuclear proton-capture reactions within this network. As such, this study provides a baseline for understanding the nuclear pathways governing aluminium production and offers a foundation for future investigations that incorporate detailed stellar structure, transport processes, and observational constraints.

- For most stars, the difference between independent determinations of εAl27 is ≤ 0.05 dex (e.g., HD7374: 5.27 vs. 5.26; HD193452: 5.65 vs. 5.65), with a few cases showing slightly larger deviations of ≈ 0.05 – 0.10 dex (e.g., HD11753: 5.02 vs. 4.80; HD89822: 4.82 vs.4.77). The absence of a systematic offset between these measurements suggests that the observational abundances are internally consistent within expected uncertainties. These uncertainties likely arise from factors such as line blending, sensitivity to stellar parameters, and non-LTE effects.
- The observed average value of εAl27≈5.10, spanning a range of 4.43–5.97, reflects the diversity of stellar properties within the sample, including variations in metallicity and physical conditions. While the present parametric model does not attempt to reproduce these values directly, the overall agreement in trends supports the view that proton-capture processes within the Ne-Al network can contribute to the underlying production of Al27 in stellar interiors.
- The results demonstrate that the extended Ne-Al chain can efficiently produce ^27^Al within the explored temperature regime. The calculated average aluminium abundance, εAl27≈0.85, together with a mean absolute deviation of ~0.40 dex, indicates that the model captures general abundance trends while retaining a moderate level of dispersion. This spread reflects variations arising from differences in thermodynamic conditions, initial compositions, and reaction rate sensitivities.
- The analysis further shows that, with updated reaction rate constants (Iliadis et al. (2010)), the branching ratios of ^23^Na remain below unity, thereby inhibiting the establishment of a fully closed Ne-Al cycle (Rowland et al. (2004)). Consequently, nucleosynthesis proceeds through advancing reaction chains rather than cyclic equilibrium, emphasizing the importance of individual reaction pathways and branching points in determining isotopic abundances.
- A comparison with representative spectroscopic abundance data (e.g., Smith (1993)) yields a strong correlation coefficient of ~ 0.85, suggesting that the model reproduces general abundance trends despite its simplified assumptions. The relatively small deviations (≲ 0.10 dex) between independent observational determinations indicate that measurement uncertainties—such as line blending, sensitivity to stellar parameters, and non-LTE effects—also contribute to the observed scatter. The absence of a systematic offset between different determinations further supports the internal consistency of the observational data.
- It is important to emphasize that the present work is based on a single-zone nuclear reaction network and does not include a self-consistent stellar evolution model. In particular, (i) no coupling between nuclear burning and stellar structure is included, (ii) transport processes such as convection, rotation, or diffusion are not modeled, and (iii) surface abundances cannot be directly inferred from the results. Therefore, the calculated abundances should be interpreted as indicative of intrinsic nuclear reaction trends and sensitivities rather than as direct predictions of stellar surface compositions.
- Deviations between the present results and spectroscopically derived abundances may arise from additional physical processes not included in the current framework. These include (i) the role of molecular weight gradients in regulating shear instabilities, (ii) the influence of magnetic fields on the transport of chemical species and angular momentum, and (iii) the impact of stellar mass loss on surface abundance evolution. The omission of these effects represents an inherent limitation of the present model and should be considered when interpreting the results.
- Nuclear reaction rate constants, which play a crucial role in determining nucleosynthetic yields, remain a significant source of uncertainty. Since many relevant reactions occur at energies far below the Coulomb barrier, their cross sections are difficult to measure directly. Indirect methods—such as the Trojan Horse method, Coulomb breakup, and the Asymptotic Normalization Coefficient technique (Descouvemont (2020))-provide important constraints and are essential for improving the reliability of reaction rates and abundance predictions.

The present work is based on a single-zone nuclear reaction network and does not include a self-consistent stellar evolution model. In particular,(i) no coupling between nuclear burning and stellar structure is included, (ii) transport processes such as convection, rotation, or diffusion are not modeled, and (iii) surface abundances cannot be directly inferred from the results. Therefore, the calculated abundances should be interpreted as indicative of intrinsic nuclear reaction trends and sensitivities rather than as direct predictions of stellar surface compositions. Deviations between the present results and abundances derived from spectroscopic analyses (e.g., Smith (1993)) may arise from additional physical processes that are not included in the current framework. These include (i) the role of molecular weight gradients in regulating shear instabilities, (ii) the influence of magnetic fields on the transport of chemical species and angular momentum, and (iii) the impact of stellar mass loss on the evolution of surface abundances. The omission of these effects represents an inherent limitation of the present model and should be taken into account when interpreting the results in an astrophysical context.

The present study highlights the broader implications of the extended Ne-Al chain for the nucleosynthetic pathways of massive stars. Despite persisting uncertainties in thermonuclear reaction rates, our results indicate that this chain plays a crucial role in shaping stellar chemical patterns. A deeper understanding of its operation not only refines stellar evolution models but also contributes to the interpretation of chemical enrichment in stellar populations and galaxies. In particular, our calculations show that the equilibrium abundance of Al27, evaluated under LTE conditions for a stellar core density of 100 g/cc across the temperature range 0.02 × 10^9^–0.1 × 109 K, maintains a strong correlation (0.85) with spectroscopic values reported in Smith (1993) supporting the relevance of the extended Ne-Al chain to the chemical evolution of massive stars as well.

## Figures and Tables

**Fig. 2:**
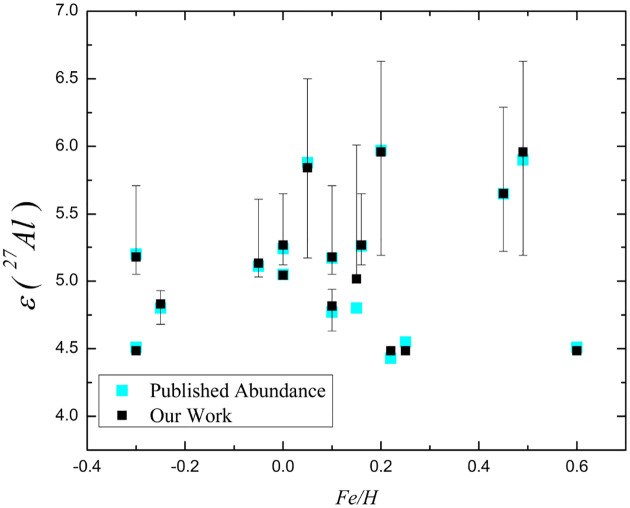
A comparative view of calculated and observed values of abundances of those selected stars of Table 6 are shown. In the fig, the cyan coloured squares are values obtained from the literature Smith (1993) and the solid black squares are the values, calculated from this work.

**Table 1:** The branching ratio $\left(B_{r}=\frac{N_{A}<\sigma v{>}_{p,\gamma }}{N_{A}<\sigma v{>}_{p,\alpha }}\right)$ at various temperatures in units of $T_{9}$. The rate constants for Na23 are taken from Iliadis et al. (2010)

$T_{9}$	BrNa23
0.02	5.95E-03
0.04	6.86E-03
0.06	1.13E-01
0.08	1.15E-01
0.10	6.63E-02

**Table 2:** The (p,γ) lifetimes $\tau_{p}(\text{s})$ for various nuclear reactions. The density in units is 100g/cc

Reaction	$T_{9}$	$N_{A}\langle \sigma v\rangle$	$\tau_{p}$
Ne20(p,γ)Na21	0.02	3.07E-21	4.65E+18
	0.04	2.73E-15	5.23E+12
	0.06	1.98E-12	7.22E+09
	0.08	1.23E-10	1.16E+08
	0.1	2.29E-09	6.24E+06
Ne21(p,γ)Na22	0.02	5.36E-22	2.67E+19
	0.04	4.51E-13	3.17E+10
	0.06	2.88E-08	4.96E+05
	0.08	6.45E-06	2.21E+03
	0.1	1.54E-04	9.28E+01
Ne22(p,γ)Na23	0.02	1.96E-16	7.29E+13
	0.04	2.02E-12	7.07E+09
	0.06	3.71E-11	3.85E+08
	0.08	3.84E-10	3.72E+07
	0.1	9.18E-09	1.56E+06
Na23(p,γ)Mg24	0.02	3.32E-24	4.30E+21
	0.04	1.88E-17	7.60E+14
	0.06	9.88E-13	1.45E+10
	0.08	5.01E-10	2.85E+07
	0.1	2.22E-08	6.44E+05
Mg24(p,γ)Al25	0.02	4.00E-26	3.57E+23
	0.04	5.01E-19	2.85E+16
	0.06	1.56E-13	9.16E+10
	0.08	3.08E-09	4.64E+06
	0.1	1.09E-06	1.31E+04
Mg25(p,γ)Al26g	0.02	4.01E-20	3.56E+17
	0.04	2.89E-13	4.94E+10
	0.06	6.37E-11	2.24E+08
	0.08	1.71E-09	8.35E+06
	0.1	1.62E-08	8.82E+05
Mg26(p,γ)Al27	0.02	6.13E-22	2.33E+19
	0.04	6.80E-15	2.10E+12
	0.06	3.35E-11	4.26E+08
	0.08	3.03E-09	4.71E+06
	0.1	5.66E-08	2.52E+05

**Table 3:** The (p,α) lifetimes $\tau_{p}(\text{s})$ for various nuclear reactions. The density in units is 100g/cc

Reaction	$T_{9}$	$N_{A}\langle \sigma v\rangle$	$\tau_{p}$
Ne20(p,α)17F	0.02	4.50E-50	3.18E+47
	0.04	1.77E-49	8.08E+46
	0.06	6.94E-49	2.06E+46
	0.08	2.73E-48	5.24E+45
	0.1	1.07E-47	1.34E+45
Ne21(p,α)18F	0.02	1.00E-99	1.43E+97
	0.04	1.00E-99	1.43E+97
	0.06	1.00E-99	1.43E+97
	0.08	1.00E-99	1.43E+97
	0.1	1.00E-99	1.43E+97
Ne22(p,α)19F	0.02	1.00E-99	1.43E+97
	0.04	1.00E-99	1.43E+97
	0.06	1.00E-99	1.43E+97
	0.08	1.00E-99	1.43E+97
	0.1	1.00E-99	1.43E+97
Na23(p,α)20 Ne	0.02	5.58E-22	2.56E+19
	0.04	2.74E-15	5.21E+12
	0.06	8.78E-12	1.63E+09
	0.08	4.37E-09	3.27E+06
	0.1	3.35E-07	4.26E+04
Mg24(p,α)Na21	0.02	1.29E-47	1.11E+45
	0.04	2.80E-47	5.10E+44
	0.06	6.10E-47	2.34E+44
	0.08	1.33E-46	1.07E+44
	0.1	2.90E-46	4.93E+43
Mg25(p,α)Na22	0.02	1.36E-60	1.05E+58
	0.04	1.25E-51	1.14E+49
	0.06	4.03E-46	3.54E+43
	0.08	4.60E-44	3.11E+41
	0.1	5.25E-42	2.72E+39
Al27(p,α)Mg24	0.02	3.07E-25	4.65E+22
	0.04	1.50E-16	9.52E+13
	0.06	1.38E-13	1.04E+11
	0.08	3.84E-12	3.72E+09
	0.1	4.34E-11	3.29E+08

**Table 4:** The (p,n) lifetimes $\tau_{p}(\text{s})$ for various nuclear reactions. The density in units is 100g/cc

Reaction	$T_{9}$	$N_{A}\langle \sigma v\rangle$	$\tau_{p}$
Ne20(p,n)Na20	0.02	3.02E-48	4.73E+45
	0.04	2.31E-40	6.18E+37
	0.06	1.62E-35	8.83E+32
	0.08	1.04E-33	1.38E+31
	0.1	6.63E-32	2.15E+29
Ne21(p,n)Na21	0.02	8.17E-48	1.75E+45
	0.04	6.33E-40	2.26E+37
	0.06	4.47E-35	3.20E+32
	0.08	2.87E-33	4.98E+30
	0.1	1.84E-31	7.75E+28
Ne22(p,n)22Na	0.02	1.00E-99	1.43E+97
	0.04	1.00E-99	1.43E+97
	0.06	1.00E-99	1.43E+97
	0.08	1.00E-99	1.43E+97
	0.1	1.00E-99	1.43E+97
Na23(p,n)23Mg	0.02	2.79E-66	5.12E+63
	0.04	4.05E-65	3.53E+62
	0.06	5.88E-64	2.43E+61
	0.08	8.53E-63	1.67E+60
	0.1	1.24E-61	1.15E+59
Mg25(p,n)25Al	0.02	4.23E-52	3.38E+49
	0.04	3.87E-43	3.69E+40
	0.06	1.25E-37	1.14E+35
	0.08	1.43E-35	1.00E+33
	0.1	1.63E-33	8.79E+30

**Table 5:** The estimated abundance by mass fraction of Al27 due to uncertainty in the reaction rate constants with low, medium (recommended) and the high rate constant with respect to hydrogen mass fraction $\left(X_{H}\right)$

$T_{9}$	Low	Medium	High
$X_{H}=0.60$			
0.02	1.56E-07	1.55E-07	1.55E-07
0.04	1.59E-07	9.34E-08	1.31E-07
0.06	1.59E-07	2.12E-07	7.23E-07
0.08	2.33E-07	1.60E-06	7.82E-06
0.1	2.33E-07	6.29E-07	2.75E-06
$X_{H}=0.62$			
0.02	1.56E-07	1.55E-07	1.55E-07
0.04	1.57E-07	9.53E-08	1.31E-07
0.06	1.59E-07	1.97E-07	5.99E-07
0.08	2.28E-07	1.32E-06	6.29E-06
0.1	2.29E-07	5.45E-07	2.24E-06
$X_{H}=0.64$			
0.02	1.56E-06	1.55E-07	1.55E-07
0.04	1.57E-07	9.78E-08	1.33E-07
0.06	1.59E-07	1.82E-07	4.75E-07
0.08	2.24E-07	1.04E-06	4.77E-06
0.1	2.24E-07	4.62E-07	1.73E-06
$X_{H}=0.66$			
0.02	1.56E-07	1.55E-07	1.55E-07
0.04	1.58E-07	1.01E-07	1.34E-07
0.06	1.58E-07	1.67E-07	3.52E-07
0.08	2.20E-07	7.64E-07	3.25E-06
0.1	2.19E-07	3.78E-07	1.23E-06
$X_{H}=0.68$			
0.02	1.56E-07	1.55E-07	1.55E-07
0.04	1.57E-07	1.08E-07	1.73E-08
0.06	1.58E-07	1.54E-07	2.31E-07
0.08	2.16E-07	4.87E-07	1.72E-06
0.1	2.13E-07	2.94E-07	7.17E-07

**Table 6:** The elemental abundance comparison for Al27

Stars	[Fe/H]	εAl27 ^a^	εAl27 ^b^	$X_{H},T_{9}$
HD7374	0.16	5.27	5.26	0.1, 0.68
HD11753	0.15	5.02	4.8	0.02, 0.64
HD27295	0.1	5.18	5.17	0.06, 0.60
HD33904	−0.05	5.13	5.11	0.06, 0.62
HD58661	−0.3	5.18	5.2	0.06, 0.60
HD77350	0.2	5.96	5.97	0.08, 0.62
HD78316	0.22	4.48	4.43	0.04, 0.7
HD79158	0.49	5.96	5.9	0.08, 0.62
HD89822	0.1	4.82	4.77	0.04, 0.64
HD141556	0.05	5.84	5.88	0.08, 0.64
HD143807	0	5.04	5.05	0.02, 0.60
HD144206	−0.3	4.48	4.51	0.04, 0.7
HD145389	0	5.27	5.24	0.1, 0.68
HD149121	0.25	4.48	4.55	0.04, 0.7
HD172044	−0.25	4.83	4.8	0.04, 0.68
HD190229	0.6	4.48	4.51	0.04, 0.7
HD193452	0.45	5.65	5.65	0.1, 0.6

a (Smith (1993)) and

b refers to our works. [Fe/H] values are taken from SIMBAD Astronomical Database.

## Data Availability

There is no research data associated with this manuscript outside of the submitted files.
